# Clinical indicators for the incidence of postoperative ileus after elective surgery for colorectal cancer

**DOI:** 10.1186/s12893-021-01093-7

**Published:** 2021-02-11

**Authors:** Yosuke Namba, Yuzo Hirata, Shoichiro Mukai, Sho Okimoto, Seiji Fujisaki, Mamoru Takahashi, Toshikatsu Fukuda, Hideki Ohdan

**Affiliations:** 1 Department of Surgery, Chugoku Rosai Hospital, 1-5-1 Hiro-Tagaya, Kure, 737-0193 Japan; 2 Department of Surgery, Saiseikai Hiroshima Hospital, 2-3-10 Kitashinchi, Saka-machi, Aki-gun, Hiroshima, 731-4311 Japan; 3 Department of Gastroenterological and Transplant Surgery Applied Life Sciences, Institute of Biomedical and Health Sciences, Hiroshima University, 1-2-3 Kasumi, Minami-ku, Hiroshima, Japan

**Keywords:** Postoperative ileus, Intraoperative in–out balance, Performance status, Elective surgery, Colorectal cancer

## Abstract

**Background:**

The occurrence of postoperative ileus leads to increased patient morbidity, longer hospitalization, and higher healthcare costs. No clear policy on postoperative ileus prevention exists. Therefore, we aim to evaluate the clinical factors involved in the development of postoperative ileus after elective surgery for colorectal cancer.

**Methods:**

We retrospectively analyzed patients who underwent elective surgery involving bowel resection with or without re-anastomosis for colon cancer between April 2015 and March 2020. The primary readout was the presence or absence of postoperative ileus. Univariate and multivariate analyses were used to identify pre- and intraoperative risk factors, and the incidence of postoperative ileus was assessed using independent factors.

**Results:**

Postoperative ileus occurred in 48 out of 356 patients (13.5%). In multivariate analysis, male sex poor performance status, and intraoperative in–out balance per body weight were independently associated with postoperative ileus development. The incidence of postoperative ileus was 2.5% in the cases with no independent factors; however, it increased to 36.1% when two factors were observed and 75.0% when three factors were matched.

**Conclusions:**

We discovered that male gender, poor performance status, and intraoperative in–out balance per body weight were associated with the development of postoperative ileus. Of these, intraoperative in–out balance per body weight is a controllable factor. Hence it is important to control the intraoperative in–out balance to lower the risk for postoperative ileus.

## Background

Prolonged postoperative ileus (POI) occurs after gastrointestinal and other types of surgery, and its incidence rate is reported to range between 10 and 30% following a major abdominal surgery [1–5]. The characteristic features of POI are bowel dysfunction and decreased motility, resulting in ineffective passage of intestinal contents [6, 7]. POI symptoms include abdominal distension, decreased or bowel sounds, constipation, and inability to advance oral intake. Persistence of these symptoms causes dehydration, electrolyte imbalance, pneumonia, and sepsis. The recovery from an abdominal surgery such as colectomy usually takes 3 to 5 days [8]. However, the occurrence of POI leads to longer hospitalization, increased patient morbidity, and higher healthcare costs [8–12]. Strong surgical stress and inflammation, physical manipulation of the bowel, and the use of anesthetics and analgesics are thought to cause POI, but its exact pathogenesis remains poorly understood [13, 14].

Numerous preventing measures and treatments for POI have been tested in randomized controlled trials. The Enhanced Recovery after Surgery (ERAS) group proposed several preventing measures for POI, including no bowel preparation, reduction of preoperative fasting period, laparoscopic approach, avoidance of abdominal drains, limitation of intravenous fluids, and immediate removal of the nasogastric tube and bladder catheter [15–18]. We are starting to understand the mechanisms of POI prevention, but no clear guidelines and policies have been established to date.

Hence, this study aimed to evaluate the clinical factors involved in the development of postoperative ileus after elective surgery for colorectal cancer.

## Methods

### Study population

This was a single-center retrospective cohort study on patients who underwent an open or laparoscopic colorectal surgery at the Chugoku Rosai Hospital (Kure, Japan). Elective colorectal surgeries involving bowel resection with or without anastomosis for colon cancer performed between April 2015 and March 2020 were included. Exclusion criteria for this study included the age of less than 18 years, cases of emergency surgery, surgeries with non-abdominal approach, and reoperations due to anastomotic leak.

The following risk factors for POI were assessed: patient background and clinical and surgical factors. Patient background data included age, sex, body mass index, performance status (PS), brain disease, heart disease, ventilation disorder, brinkman index, and Charlson comorbidity index. Clinical and surgical factors included preoperative blood test results, preoperative intestinal decompression, presence or absence of anastomosis, tumor occupation site, extent of bleeding, operation time, and intraoperative in–out balance per body weight (IOB/BW). Intraoperative in–out balance was defined as the volume of fluid or blood transfusion minus the volume of urine and bleeding. The following items of the ERAS protocol were followed: preoperative counseling, prevention of nausea and vomiting, antimicrobial prophylaxis and skin preparation, preoperative fasting, intraoperative hypothermia prevention, no postoperative nasogastric tube, postoperative mechanical thromboprophylaxis, postoperative glycemic control, postoperative resumption of oral intake, and early mobilization [18].

Patients gave their informed consent for the use of their data. The study was approved by the Medical Ethics Committee of Chugoku Rosai Hospital (NO. 2020-21). All methods were performed in accordance with the relevant guidelines and regulations.

### Definition of postoperative ileus

The primary readout was the presence or absence of POI, and POI was defined based on a meta-analysis by Vather et al. [2]. According to Vather et al., POI occurs when two or more of the following five criteria are met on or after the fourth postoperative day without improvement: (i) nausea and vomiting; (ii) inability to tolerate oral food intake for at least 24 h; (iii) absence of flatus for 24 h; (iv) abdominal distension; and (v) radiological evidence of ileus.

### Statistical analysis

Descriptive statistics for categorical variables are reported as absolute numbers and continuous variables as mean (± standard deviation) values. Categorical variables were analyzed with Fisher’s exact test, while continuous variables were calculated using Student t test. Statistical significance was set at *P* < 0.05. In continuous variables with *P* < 0.05, as indicated by univariate analysis, and cutoff values were specified using the receiver operating characteristic curve analysis. Multivariate logistic regression was performed using factors with *P* < 0.05 in the univariate analysis as the independent variable and the presence or absence of POI as the dependent variable. For independent variables expected to have strong correlations, internal correlations were calculated by Spearman’s rank correlation test to avoid multicollinearity. If the correlation was very strong, one of the factors was excluded. Data were analyzed using the Statistical Package for Social Sciences (SPSS 22.0, Inc., Chicago, IL).

## Results

Between April 2015 and March 2020, 377 patients who underwent open or laparoscopic colorectal surgery were assessed for eligibility. There were 21 patients who met the exclusion criteria, among them 11 patients underwent an emergency surgery, two patients were operated with a non-abdominal approach, and eight patients underwent a reoperation due to anastomotic leak.

POI occurred in 48 of the 356 patients analyzed (13.5%). Baseline demographics are shown in Table 1. POI was more frequent in male patients (72.9% vs 51.2%, *P* < 0.01). POI incidence was 11.6% in patients with a PS of 0 or 1 and 27% in patients with a PS of 2 or more (*P* < 0.01).

**Table 1:** Baseline demographics

	No-POI group n = 308 (86.5%)	POI group n = 48 (13.5%)	*p* value
Age, mean (SD), y	74.5 ± 10.8	72.7 ± 9.38	0.29
Male gender, n (%)	158 (51.2)	35 (72.9)	< 0.01
Body mass index, mean (SD), kg/m^2^	23.1 ± 4.47	24.3 ± 4.95	0.07
Performance status (2 - 4), n (%)	33 (11.6)	13 (27.0)	< 0.01
Brain disease, n (%)	39 (12.6)	9 (18.7)	0.25
Heart disease, n (%)	48 (15.5)	11 (22.9)	0.21
Ventilation disorder, n (%)	69 (22.4)	10 (20.8)	1
Brinkman index >400, n (%)	85 (27.5)	18 (37.5)	0.17
Charlson comorbidity index, mean (SD), points	0.97 ± 1.70	1.29 ± 1.97	0.23

Categorical variables were analyzed using Fisher’s exact test, and continuous variables were analyzed using Student t test. *POI* postoperative ileus

The clinical and surgical factors are displayed in Table 2. The following factors significantly differed between the POI and non-POI groups: the extent of bleeding (477.0 ± 762.5 ml vs 169.4 ± 366.5 ml, *P* < 0.01), operation time (402.2 ± 132.2 min vs 307.3 ± 95.8 min, *P* < 0.01), and IOB/BW (42.8 ± 18.7 ml vs 33.7 ± 12.5 ml, *P* < 0.01).

**Table 2:** Clinical and surgical characteristics

	No-POI group n = 308 (86.5%)	POI group n = 48 (13.5%)	*p* value
Neut/Lymph ratio, mean (SD), %	3.18 ± 2.25	3.25 ± 2.14	0.84
CRP, mean (SD), mg/dL	1.24 ± 2.92	1.98 ± 4.42	0.13
Cholinesterase, mean (SD), IU/L	255.3 ± 77.0	253.7 ± 79.2	0.89
Albumin, mean (SD), g/dL	3.75 ± 0.64	3.65 ± 0.66	0.34
eGFR, mean (SD), mL/minutes/1.73 m^2^	70.7 ± 19.4	74.3 ± 32.3	0.29
Preoperative intestinal decompression, n (%)	64 (20.7)	9 (18.7)	0.84
Laparotomy, n (%)	76 (24.6)	18 (37.5)	0.07
Extent of bleeding, mean (SD), mL	169.4 ± 366.59	477.0 ± 762.5	< 0.01
Operation time, mean (SD), minutes	307.3 ± 95.8	402.2 ± 132.2	< 0.01
IOB/BW, mean (SD), mL/kg	33.7 ± 12.5	42.8 ± 18.7	< 0.01
Epidural anesthesia, n (%)	261 (84.70)	39 (81.20)	0.52
Anastomosis, n (%)	281 (91.2)	40 (83.3)	0.11
Tumor occupation site			
Right colon, n (%)	106 (34.40)	10 (20.80)	0.69
Left colon, n (%)	120 (38.90)	24 (50.0)	0.15
Rectum, n (%)	82 (26.60)	14 (29.10)	0.72

Categorical variables were analyzed using Fisher’s exact test, and continuous variables were analyzed using Student t test

*POI* postoperative ileus, *Neut* neutrophil, *Lymph* lymphocytes, *CRP* C-reactive protein, *eGFR* estimated glomerular filtration rate, *IOB/BW* intraoperative in-out balance per body weight

Internal correlations were calculated for extent of bleeding, operation time, and intraoperative IOB/BW, which were considered to have strong clinical correlation. Strong correlations were observed between extent of bleeding and operation time (r = 0.38, *P* < 0.01), extent of bleeding and intraoperative IOB/BW (r = 0.42, *P* < 0.01), and operation time and intraoperative IOB/BW (r = 0.48, *P* < 0.01), respectively. We excluded extent of bleeding and operation time, because only intraoperative IOB/BW was clear for reasons associated with POI.

**Fig. 1:**
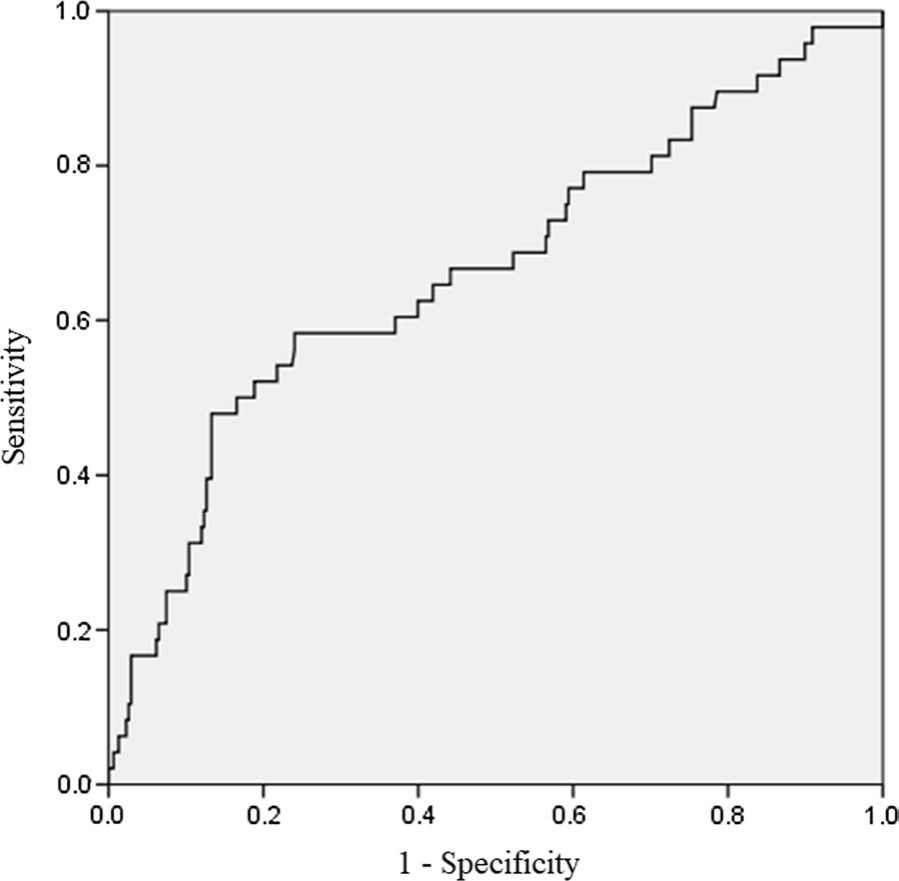
Receiver operating characteristic curves for intraoperative in–out balance per body weight. The area under the curve (AUC) was 0.66 (95% confidence interval [CI], 0.57–0.75), and the cutoff value was 46.3 ml/kg (sensitivity: 0.47, specificity: 0.86)

The cutoff value of the intraoperative IOB/BW was 46.3 ml/kg (sensitivity: 0.47, specificity: 0.86, AUC: 0.66) (Fig. 1). In the multivariate logistic regression analysis, male sex, poor PS, and intraoperative IOB/BW were independently associated with POI development (OR = 2.98 [95% CI, 1.45–6.12], *P* < 0.01; OR = 4.13 [95% CI, 1.83–9.30], *P* < 0.01; OR = 6.56 [95% CI, 3.19–13.5], *P* < 0.01, respectively) (Table 3).

**Table 3.** Multivariate logistic regression of risk factors associated of POI

	Odds Ratio (95% CI)	*p *value
Male gender	2.98 (1.45–6.12)	< 0.01
Performance status (2–4)	4.13 (1.83–9.30)	< 0.01
IOB/BW ≥ mL/kg	6.56 (3.19–13.5)	< 0.01

*POI* postoperative ileus, *IOB/BW* intraoperative in-out balance per body weight

**Fig. 2:**
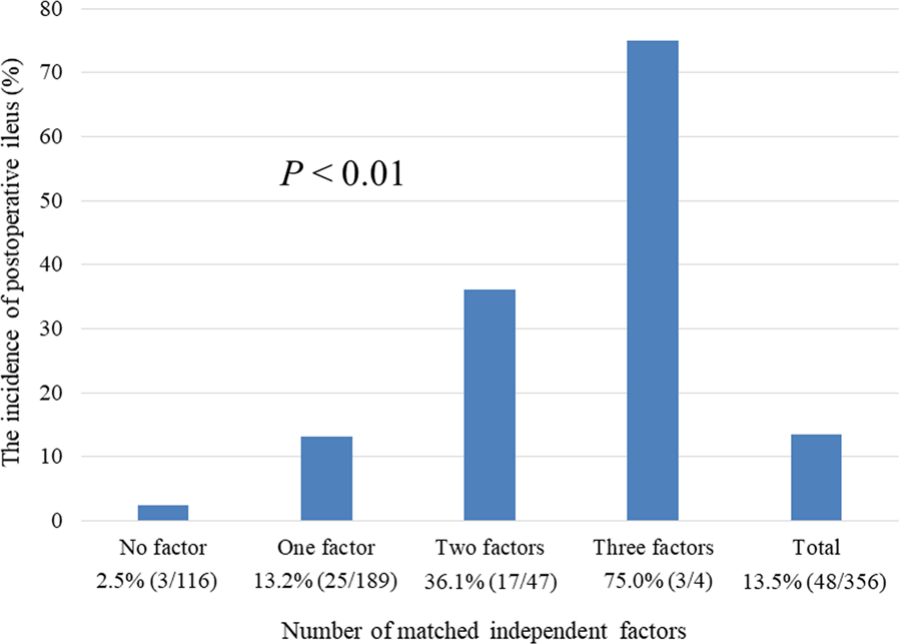
Number of matched independent factors and the incidence of postoperative ileus. The independent factors included are poor performance status (0 or 1) and intraoperative in–out balance per body weight. The incidence of anastomotic leakage is 2.5% when all factors are absent; however, it increases to 36.1% with two factors and to 75.0% with three factors

The incidence of POI was assessed using three factors: male sex, poor PS, and intraoperative IOB/BW (Fig. 2). The incidence of POI was 2.5% in the cases with no independent factors and 13.2% when one factor was observed, which was almost the same as the overall incidence. However, it increased to 36.1% when two factors were observed and 75.0% when three factors were matched.

## Discussion

This study identified clinical factors associated with POI development after elective surgery for colorectal cancer. Male sex, poor PS, and intraoperative IOB/BW were independently associated with POI development.

The prevention of POI is an important part of preoperative management as POI leads to increased patient morbidity, length of hospital stay, and healthcare costs [8–12]. Although some studies reported that factors such as infection, surgical stress response, physical manipulation of the bowel, nutritional delay, and postoperative complications adversely affect intestinal motility and may lead to prolonged POI [19, 20], the clear pathogenesis of POI remains incompletely understood. Grass et al. suggested the effects of intestinal edema due to perioperative fluid overload [21]. Few reports have shown the association between perioperative fluid overload and POI, and this study focused on intraoperative IOB / BW and examined other factors involved in POI.

In our study, male sex, poor PS, and intraoperative in–out balance were associated with POI development. Patients with poor PS tend to spend prolonged periods resting in bed. Holte et al. reported that lengthy bed rest is associated with POI, whereas faster recovery prevents POI [22]. However, there is no evidence that POI can be prevented by increasing ambulation [10, 23]. POI may occur in patients with poor PS, such as bedridden patients, due to small feedings and poor pain control (due to difficulty expressing pain), not because of decreased ambulation. Some studies reported that small feedings and pain contribute to the occurrence of POI [24, 25]. In the present study, the amount of feedings and pain have not been evaluated and thus are subject to future studies. There is a strong evidence to report that fluid overload may contribute to POI development [26]. It has been reported that splanchnic edema due to fluid overload may result in decreased mesenteric blood flow and increased abdominal pressure, which in turn elicits tissue hypoxia and ultimately leads to ileus and anastomotic healing [27]. Lobo et al. described the critical role of perioperative fluid balance and avoidance of interstitial fluid overload in the elective setting [28], and Grass et al. also reported that fluid overload is a risk factor for POI [21]. These reports supported our findings that intraoperative in–out balance for each body weight was associated with the incidence of POI. Several reports showed that male sex is associated with the incidence of POI [3, 29, 30]. Huskisson reported that men are more likely to feel pain and experience increased catecholamine release; however, the exact mechanism is unknown [29].

The incidence of POI was assessed using three factors. In the absence of these three factors, the incidence of POI was only 2.5%; however, it increased to 36.1% in the presence of two factors and to 75.0% in the presence of three factors. Of these factors, only intraoperative IOB/BW could be controlled intraoperatively. Intraoperative IOB/BW strongly influences the incidence of POI and is a factor that can be easily adjusted; hence, more careful control is required. Special attention is needed in patients with poor PS and male sex.

The present study has several limitations. First, it was conducted at a single center and was retrospective. Therefore, patient selection bias may have been present. Additionally, this study did not evaluate the degree of pain. Poor PS and male sex may be associated with pain and need to be reassessed with this factor added in the future. It is necessary to repeat similar studies and show reproducibility. Further studies with larger cohorts of patients will be necessary to provide additional support for these findings.

## Conclusions

We discovered that male sex, poor PS, and intraoperative IOB/BW were associated with the development of POI. Of these, intraoperative IOB/BW is a controllable factor; hence, it is important to control the intraoperative in–out balance to lower the risk for POI.

## Data Availability

All data is contained within the manuscript. The datasets used and analyzed during the current study are available from the corresponding author on reasonable request.

## Abbreviations

POI: Postoperative ileus
PS: Performance status
IOB/BW: In–out balance per body weight

## References

[1] Vather R, Josephson R, Jaung R, Robertson J, Bissett I (2015). Development of a risk stratification system for the occurrence of prolonged postoperative ileus after colorectal surgery: a prospective risk factor analysis. Surgery.

[2] Vather R, Trivedi S, Bissett I (2013). Defining postoperative ileus: results of a systematic review and global survey. J Gastrointest Surg.

[3] Chapuis PH, Bokey L, Keshava A, Rickard MJFX, Stewart P, Young CJ (2013). Risk factors for prolonged ileus after resection of colorectal cancer: an observational study of 2400 consecutive patients. Ann Surg.

[4] Moghadamyeghaneh Z, Hwang GS, Hanna MH, Phelan M, Carmichael JC, Mills S (2016). Risk factors for prolonged ileus following colon surgery. Surg Endosc.

[5] Kim MJ, Min GE, Yoo KH, Chang S-G, Jeon SH (2011). Risk factors for postoperative ileus after urologic laparoscopic surgery. J Korean Surg Soc.

[6] Luckey A, Livingston E, Taché Y (2003). Mechanisms and treatment of postoperative ileus. Arch Surg.

[7] Resnick J, Greenwald DA, Brandt LJ (1997). Delayed gastric emptying and postoperative ileus after nongastric abdominal surgery: part II. Am J Gastroenterol.

[8] Schuster TG, Montie JE (2002). Postoperative ileus after abdominal surgery. Urology.

[9] Chang SS, Baumgartner RG, Wells N, Cookson MS, Smith JA (2002). Causes of increased hospital stay after radical cystectomy in a clinical pathway setting. J Urol.

[10] Mattei P, Rombeau JL (2006). Review of the pathophysiology and management of postoperative ileus. World J Surg.

[11] Mythen MG (2005). Postoperative gastrointestinal tract dysfunction. Anesth Analg.

[12] Springer JE, Elkheir S, Eskicioglu C, Doumouras AG, Kelly S, Yang I (2018). The effect of simethicone on postoperative ileus in patients undergoing colorectal surgery (SPOT), a randomized controlled trial. Int J Surg.

[13] Bragg D, El-Sharkawy AM, Psaltis E, Maxwell-Armstrong CA, Lobo DN (2015). Postoperative ileus: recent developments in pathophysiology and management. Clin Nutr.

[14] Venara A, Neunlist M, Slim K, Barbieux J, Colas PA, Hamy A (2016). Postoperative ileus: pathophysiology, incidence, and prevention. J Visc Surg.

[15] Wang H, Zhu D, Liang L, Ye L, Lin Q, Zhong Y (2015). Short-term quality of life in patients undergoing colonic surgery using enhanced recovery after surgery program versus conventional perioperative management. Qual Life Res.

[16] Zhuang C-L, Ye X-Z, Zhang X-D, Chen B-C, Yu Z (2013). Enhanced recovery after surgery programs versus traditional care for colorectal surgery: a meta-analysis of randomized controlled trials. Dis Colon Rectum.

[17] Nygren J, Thacker J, Carli F, Fearon KCH, Norderval S, Lobo DN (2013). Guidelines for perioperative care in elective rectal/pelvic surgery: Enhanced Recovery After Surgery (ERAS(®)) Society recommendations. World J Surg.

[18] Gustafsson UO, Scott MJ, Schwenk W, Demartines N, Roulin D, Francis N (2013). Guidelines for perioperative care in elective colonic surgery: Enhanced Recovery After Surgery (ERAS(®)) Society recommendations. World J Surg.

[19] Türler A, Moore BA, Pezzone MA, Overhaus M, Kalff JC, Bauer AJ (2002). Colonic postoperative inflammatory ileus in the rat. Ann Surg.

[20] Artinyan A, Nunoo-Mensah JW, Balasubramaniam S, Gauderman J, Essani R, Gonzalez-Ruiz C (2008). Prolonged postoperative ileus-definition, risk factors, and predictors after surgery. World J Surg.

[21] Grass F, Lovely JK, Crippa J, Hübner M, Mathis KL, Larson DW (2020). Potential association between perioperative fluid management and occurrence of postoperative ileus. Dis Colon Rectum.

[22] Holte K, Kehlet H (2002). Postoperative ileus: progress towards effective management. Drugs.

[23] Waldhausen JH, Schirmer BD (1990). The effect of ambulation on recovery from postoperative ileus. Ann Surg.

[24] Mangesi L, Hofmeyr GJ (2002). Early compared with delayed oral fluids and food after caesarean section. Cochrane Database Syst Rev..

[25] Bauer AJ, Boeckxstaens GE (2004). Mechanisms of postoperative ileus. Neurogastroenterol Motil.

[26] Lobo DN (2009). Fluid overload and surgical outcome: another piece in the jigsaw. Ann Surg.

[27] Mayberry JC, Welker KJ, Goldman RK, Mullins RJ (2003). Mechanism of acute ascites formation after trauma resuscitation. Arch Surg (Chicago, Ill: 1960)..

[28] Lobo DN, Macafee DA, Allison SP (2006). How perioperative fluid balance influences postoperative outcomes. Best Pract Res Clin Anaesthesiol.

[29] Huskisson EC (1974). Catecholamine excretion and pain. Br J Clin Pharmacol.

[30] Ferguson JF, Patel PN, Shah RY, Mulvey CK, Gadi R, Nijjar PS (2013). Race and gender variation in response to evoked inflammation. J Transl Med.

